# Uterine pseudoaneurysm on the basis of deep infiltrating endometriosis during pregnancy-a case report

**DOI:** 10.1186/s12884-021-03753-1

**Published:** 2021-04-09

**Authors:** Tibor Andrea Zwimpfer, Cécile Monod, Katharina Redling, Heike Willi, Martin Takes, Bernhard Fellmann-Fischer, Gwendolin Manegold-Brauer, Irene Hösli

**Affiliations:** 1 Department of Obstetrics and Gynecology, University Hospital of Basel, Basel, Switzerland; 2 Department of Biomedicine, University Hospital of Basel and University Basel, Basel, Switzerland; 3 Department of Radiology and Nuclear Medicine, University Hospital of Basel, Basel, Switzerland

**Keywords:** Uterine pseudoaneurysm, Deep infiltrating endometriosis, Pregnancy

## Abstract

**Background:**

Pseudoaneurysm of the uterine artery (UPA) is a rare cause of potentially life-threatening hemorrhage during pregnancy and puerperium. It is an uncommon condition that mainly occurs after traumatic injury to a vessel following pelvic surgical intervention, but also has been reported based on underlying endometriosis. There is an increased risk of developing UPA during pregnancy. Diagnosis includes clinical symptoms, with severe abdominal pain and is confirmed by sonographic or magnetic resonance imaging (MRI). Due to its potential risk of rupture, with a subsequent hypovolemic maternal shock and high fetal mortality, an interdisciplinary treatment should be considered expeditiously.

**Case presentation:**

We present the case of a 34-year old pregnant symptomatic patient, where a large UPA was detected at 26 weeks, based on deep infiltrating endometriosis (DIE). The UPA was successfully treated by selective arterial embolization. After embolization, the pain decreased but the woman still required intravenous analgesics during follow-up. At 37 weeks she developed a sepsis from the intravenous catheter which led to a cesarean section and delivery of a healthy boy. She was discharged 10 days postpartum.

**Conclusions:**

UPA should be considered in pregnant women with severe abdominal and pelvic pain, once other obstetrical factors have been excluded. DIE might be the underlying diagnosis. It is a rare but potentially life-threatening condition for mother and fetus.

## Background

Uterine pseudoaneurysm (UPA) is a condition in which the arterial vessel wall has lost intraluminal continuity and blood accumulates between the two outer layers of the artery. It can present with severe abdominal and pelvic pain, and sonographic imaging or magnet resonance imaging (MRI) can detect a pulsatile growing mass. UPA occurs mainly after a traumatic injury of the vessel following pelvic surgical intervention, but rarely, it is based on severe endometriosis as DIE (Deep infiltrating endometriosis) [1–5]. The main causes are gynecological interventions, such a myomectomy, treatment of endometriosis, ovarian puncture or cystectomies, and obstetrical interventions, such as cesarean section, curettage, and vacuum or forceps extraction [3–9]. Furthermore, there is an increased risk of developing UPA during pregnancy.

Due to the potential risk of rupture, with subsequent hypovolemic shock of the mother and a high fetal mortality, the diagnosis of UPA in pregnancy requires urgent interdisciplinary treatment. The standard of care is a selective arterial embolization of the uterine artery by the interventional radiologist, which has a good risk-benefit profile [10, 11].

Here, we present the observation of a pregnant patient with a successfully treated symptomatic UPA that occurred in the second trimester, on the basis of a DIE in the left uterine artery and cervix.

## Case presentation

**Fig. 1:**
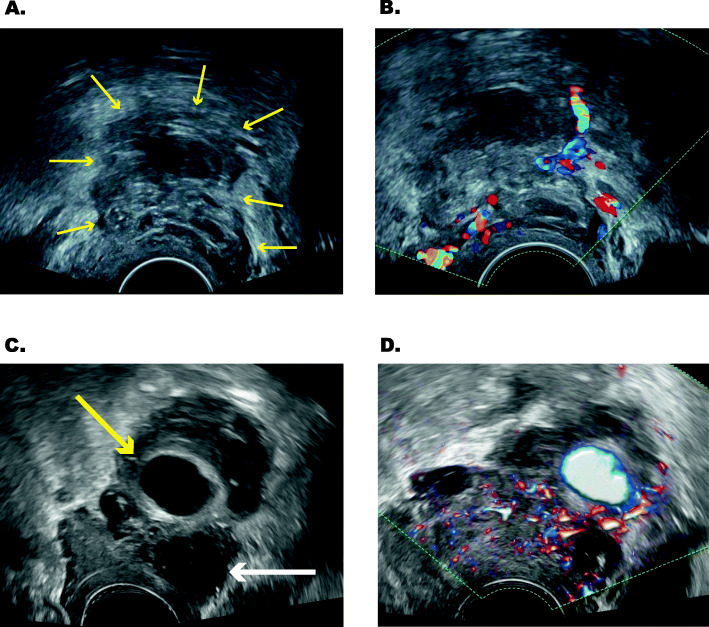
Development of the UPA over 2 weeks by ultrasound. Visit 1 with **a.** Ultrasound image of the ill-defined solid mass in the adnex at the initial presentation at 23 + 2 gestational weeks. The arrows mark the outer margins of the lesion. An endometrioma is not clearly visible. **b.** Corresponding Doppler image of the left adnexa showing moderate blood flow (Color Score 3) in the lesion. Visit 2 with **c** Ultrasound Image of the same lesion on the follow up at 25 + 2 gestational weeks. On the lower right there is an unilocular mass suggestive of endometrioma. In the center there is a pulsating vessel (UPA) of about 2 cm with most likely haematoma surrounding the UPA and **d.** corresponding Doppler image confirming blood flow in the vessel.

**Fig. 2:**
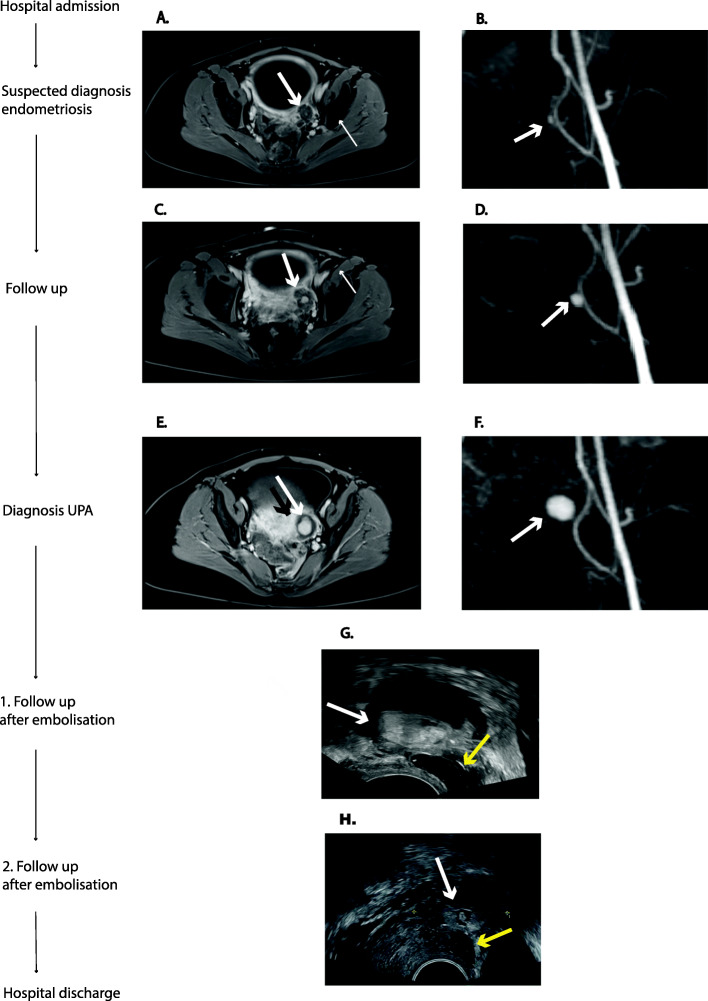
Timeline of the development of the endometriosis nodule and pseudoaneurysm of the uterine artery (UPA), from transvaginal ultrasound (TVUS) and magnetic resonance imaging (MRI). **a:** T1 weighted transverse MRI showing a lesion (2 cm × 2 cm) caudal adjacent to the left ovary, suspected to be an endometriosis nodule; **b:** Dynamic MR angiography (TWIST) showing a small process of the left uterine artery.; **c**.T1 weighted transverse MRI showing a progressing lesion (3.8 cm × 2 cm) with expansion to the rectovaginal space with **d.** a corresponding growing alteration of the left uterine artery in the TWIST.; **e.** T1 weighted transverse MRI with an identifiable UPA (2.5 cm × 1.5 cm) and the confirmation in the **f.** TWIST.; **g.** TVUS showing the UPA (left) (black arrow) and the endometriosis nodule (white arrow) one day after embolization; **h:** TVUS follow-up ultrasound a week after embolization, showing the UPA left (black arrow) and endometriois nodule (white arrow)

The 34-year old, first gravida, was admitted to our obstetrical department by ambulance at 23 + 0 weeks of gestation (WG) with progressive severe pain over 24 h in the left lower abdomen irradiating to the rectum and the vagina. The previous day, she had an unremarkable clinical and sonographic examination and a normal laboratory investigation. Her past medical history included a conization due to cervical dysplasia, dysmenorrhea and dyspareunia with suspected endometriosis, and the use of a combined oral contraceptive for 16 years prior to the current pregnancy. The pregnancy occurred spontaneously, with a single fetus. The otherwise healthy patient showed pain on palpation in the two lower abdominal quadrants: speculum examination revealed cervical ectopy and two black dots were visible at six o’clock. The vital signs were normal with an unremarkable pulse, respiratory rate, body temperature, blood pressure, and the hemoglobin was stable at 129 g/l. The laboratory tests and urine analysis showed no signs of infection. The cardiotocogram (CTG) was normal without contractions. Cervical length was 34 mm, measured by transvaginal ultrasound. Fetal sonography and Doppler studies revealed normal biometry with a fetus appropriate for gestational age at 42nd percentile, normal amniotic fluid, a posterior wall placenta without signs of hematoma and a normal uteroplacental resistance. Caudal and adjacent to the left ovary, a solid, ill-defined adnexal mass of 40x45mm and moderate blood flow was detected (Fig. 1 a, b). On MRI, the adnexal mass was seen and was suggestive of endometriosis (Fig. 2 a and b). There were no signs of intraabdominal free fluid or kidney stones.

The patient was hospitalized for monitoring and analgesic therapy. The pelvic pain persisted despite intravenous (IV) opiate therapy. Additionally, the patient complained of newly occurring dyschezia and a single episode of brown vaginal discharge. A follow-up MRI after 6 days, at 24 + 1 WG showed progression of the lesion, with expansion into the rectovaginal space (Fig. 2 c and d). The patient remained hospitalized for maternal and fetal monitoring and received continuous analgesia and steroids for fetal lung maturation induction with tocolysis as a precautionary measure. Sixteen days after hospitalization (25 + 3 WG), a pseudoaneurysm of the left uterine artery (2.5 cm × 1.5 cm) was identified by ultrasound (Fig. 1 c and d) and confirmed by MRI. (Fig. 2 e and f). An interdisciplinary team of radiologist, fetomaternal specialists, gynecologists and neonatologists concluded that, based on the early gestational age, the progression of the lesion and in view to the persistent pain, a selective embolization of the left uterine artery would be the preferred management. The neonatologist team was prepared for an emergency cesarean section in case of any complications. The intervention was carried out under local anesthesia with a retrograde access over the left common femoral artery. No digital subtraction angiography was performed in order to minimize radiation exposition. Embolization was achieved by selective occlusion of the aneurysm-feeding branch of the left uterine artery over a microcatheter by injection of 0.2 ml liquid embolic material (histoacryl/lipiodol 1:3). The estimated radiation exposure for the uterus was 0.4 mSv. Subsequently the contrast media had suspended in the UPA as indication for a sufficient occlusion. Further sequential sonographic examinations confirmed normal fetal growth and Doppler flow. The embolized UPA showed no vascularization and decreased to 2 cm × 1 cm on sonography before hospital discharge (Fig. 2 g and h). The pelvic pain improved but did not resolve completely and the patient received a peripherally inserted central venous catheter (PICC) line and, temporarily, a patient-controlled analgesia pump. After 30 days (28 + 2 WG), she was discharged to outpatient care with oral morphine in reserve and the PICC line in situ. A primary cesarean at 38 weeks was planned because of the risk of the UPA rupturing during contractions. However, the patient presented herself at 37 weeks, with sepsis of unknown etiology, a fever of 39.0 °C, tachypnea, hypotension, maternal tachycardia, a C-reactive protein of 23.8 mg/l and fetal tachycardia (200 beats per minute). An emergency cesarean delivery was done under general anesthesia with antibiotic therapy (amoxicillin and clavulanic acid) and a healthy boy was born (2670 g) with Apgar 6/7/8 and pH 7.31. Postoperatively, the patient needed intensive care (IC) for 3 days. Blood cultures were positive for *Serratia macescens* and *Streptococcus anginosus*, and according to the resistance tests, treatment was changed to carbapenem IV. The patient was discharged, together with the newborn, 10 days after the cesarean section. The infection was most likely caused by an infected PICC line, even though the results of the smear tests and cultures were unremarkable.

**Fig. 3:**
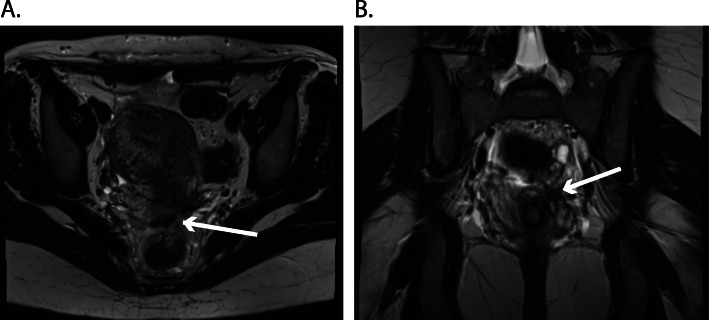
Abdominal magnetic resonance imaging (MRI) two months after the delivery **a.** T2 weighted transverse MRI and **b.** T2 weighted coronal MRI showed an endometriosis node 3x2cm (white arrow) adjacent to the septum rectovaginale with expansion to the left ovary and in close proximity to the sigmoid without infiltration according to an Enzian score A2, B1, C1

The patient presented for follow-up at 6 weeks postpartum. She had lactation amenorrhea, persisting dyschezia and newly developed hematochezia. Rectovaginal sonography and palpation identified an unchanged endometriosis node. We started suppressive therapy with Desogestrel and scheduled a colonoscopy to exclude another origin for the hematochezia and an MRI for staging. The MRI showed an endometriosis node 3x2cm adjacent to the septum rectovaginale with expansion to the left ovary and in close proximity to the sigmoid without infiltration according to an Enzian score A2, B1, C1. (Fig. 3 a and b) The restructuring operation of the symptomatic DIE is planned for 4 months after her delivery.

## Discussion

This case illustrates the difficulty to diagnose the rare entity of a UPA during pregnancy. In our case, the appearance of the lesion changed over time and finally led to the correct diagnosis with the combination of sonography and MRI. There is an increased risk of developing or diagnosing UPA during pregnancy. It is assumed that the physiological changes of the hormonal milieu and cardiovascular system, together with the pressure on the vessels promote the development of UPA [12]. Additionally, the improvement of imaging technology and the frequent ultrasounds during pregnancy increase the probability of diagnosing a UPA.

Endometriosis further increases the risk of UPA during and after pregnancy, in particular DIE [13–15]. The endometrial implants demonstrate a non-location response to hormonal stimulation. Estrogens are a proliferating factor, and the hormone withdrawal results in abortive bleeding which is associated with pain. Additionally, inflammatory cell production is stimulated, resulting in pain and adhesions. Gestagen inhibits the inflammatory reaction. In menopause, the decline of ovarian stimulation turns active endometriosis lesions inactive. Since pregnancy has a similar effect, with a decline of ovarian stimulation and increasing gestagen levels, a common assumption is that pregnancy temporarily cures endometriosis [16–18]. Recently, conflicting data demonstrates preexisting endometriosis causes pregnancy complications due to adhesions, chronic inflammation and intrusion of decidualized endometriosis [19–21]. Chronic inflammation makes the vessels more vulnerable to lacerations [22] and adhesion can increase the stress on uterine-ovarian vessels [23]. The intrusion of decidualized endometriosis can result in a perforation of the uterine-ovarian vessels and, because of persistent progesterone levels, decidualization occurs with differentiation of mesenchymal cells [24, 25]. A decrease in progesterone at the end of the third trimester of pregnancy correlates with an increased expression of inflammatory cells, proteolytic degradation of the extracellular matrix, cell death, and, finally, bleeding of the peritoneum [26, 27].

In this patient, the combination of preexisting DIE and pregnancy probably caused the UPA. The close follow-up, with ultrasound and MRI, enabled us to detect the development of the UPA from the endometriosis node during the second trimester. Previously, ruptured or unruptured UPA have been detected in pregnant patients with a known history of endometriosis or previous surgery [13–15]. Van Coppenollea et al. summarized six cases of UPA, based on previous appendectomy, cesarean section or surgically treated endometriosis [9]. Feld et al. described even a case of a hemoperitoneum, caused by a ruptured UPA based on endometriosis, but the UPA was only detected postpartum [13]. Our patient was symptom-free apart from occasional dyspareunia and dysmenorrhea because of a suspected rectovaginal endometriosis before pregnancy, as she was on hormonal contraception for 16 years until 6 months before pregnancy. We presume that the pregnancy stimulated the decidualization of the endometriosis, in particular of the deep infiltrating rectovaginal node adjacent to the UPA. The chronic inflammation, in combination with the decidualization, might have increased the stress on the uterine artery resulting in the UPA. Fortunately, the UPA was diagnosed early, and the expedited treatment preserved the pregnancy and avoided preterm delivery.

Transcatheter arterial embolization has been established as an effective technique for the management and prevention of obstetric and gynecologic hemorrhage [10, 11, 15, 28–30]. Complications of transcatheter arterial embolization are extremely uncommon when it is performed by expert interventional radiologists. Its advantages include prevention of surgical risks, high success rates, low complication rates, and no significant impact on future pregnancies and fertility [10, 11, 15, 28–30]. Three reported cases in literature [11, 14, 30] and our case suggest that successful unilateral uterine artery embolization is well tolerated by the fetus and therefore appears to be a safe and effective method to treat pseudoaneurysm during pregnancy without compromising uteroplacental perfusion. Moreover, in our case the estimated radiation exposure for the uterus was only 0.4 mSv, which is far below any critical exposure rate for the fetus.

Our patient suffered long-term from severe immobilizing pain, which was difficult to control. Furthermore, the hospitalization and treatment were physically and emotionally very stressful. This raises the question regarding early diagnosis and treatment of such cases through monitoring pregnancies of patients with endometriosis. Currently, there is no evidence that endometriosis has a significant effect on pregnancy outcome [31, 32].; however, rare cases such as our case might be encountered in pregnancy and a data base of deep infiltrating endometriosis like that available at the Kepler University Clinic together with the Foundation Endometriosis Research (SEF), and with support of the Deutsche Gesellschaft für Gynäkologie und Geburtshilfe (DGGG) will be helpful in identifying patients at risk for such a complication and provide diagnostic and treatment guidelines.

## Conclusions

UPA should be considered in pregnant women with severe abdominal and pelvic pain, once other obstetrical factors have been excluded. Endometriosis can cause UPA during and after pregnancy, in particular DIE. It is a rare but potentially life-threatening condition for the mother and fetus, as a rupture of the UPA will result in hemoperitoneum and hypovolemic, hemorrhagic shock. The standard of care in a stable situation is selective arterial embolization, which has a good risk-benefit profile. There is currently no evidence that endometriosis has a harmful effect on the pregnancy outcome, therefore, no special monitoring of conventional pregnancies for patients with endometriosis is required. Nevertheless, awareness should be raised among physicians, and similar cases should be reported to establish treatment guidelines.

## Data Availability

The data and materials analyzed during the current case report are presented within the manuscript and available from the corresponding author.

## Abbreviations

CTG: Cardiotocogram
DIE: Deep infiltrating endometriosis
IC: Intensive care
IV: Intravenous
MRI: Magnetic resonance imaging
PICC: Peripherally inserted central venous catheter
TVUS: Transvaginal ultrasound
UPA: Uterine pseudoaneurysm
WG: Weeks of gestation
